# A displaced stress fracture of the femoral neck in an adolescent female distance runner with female athlete triad: A case report

**DOI:** 10.1186/1758-2555-2-6

**Published:** 2010-03-05

**Authors:** Shinichi Okamoto, Yuji Arai, Kunio Hara, Takashi Tsuzihara, Toshikazu Kubo

**Affiliations:** 1 Dept of Orthopaedics, Graduate School of Medical Science, Kyoto Prefectural University of Medicine, Kawaramachi-Hirokoji, Kamigyo-ku, Kyoto, 602-8566, Japan; 2 Dept of Orthopaedic, Kyoto Interdisciplinary Institute Hospital of Community Medicine, Murasakino Unrinin-tyo17, Kita-ku, Kyoto, 603-8214, Japan

## Abstract

This report presents a case of a displaced stress fracture of the femoral neck in an adolescent female distance runner with amenorrhea. Both reduction and internal fixation were performed early after the injury. At 24 months postoperatively, magnetic resonance imaging and bone scintigraphy showed no positive signs of femoral head necrosis and bone union was confirmed on plain X-ray. A medical examination for the presence of the signs of the female athlete triad by checking weight, calorie intake and menstrual cycles is most important to prevent such stress fractures. Athletes as well as their coaches or parents therefore need to understand female athlete triad.

## Background

Stress fractures of the limbs frequently occur in adolescent female distance runners who develop amenorrhea, an eating disorder, and osteopenia, the so-called "female athlete triad" [1]. While most stress fractures are found in the tibia, they can rarely occur in the femoral neck. This report presents a case of a displaced stress fracture of the femoral neck in an adolescent female distance runner with "female athlete triad". To the authors' knowledge, this is the first case report that untreated female athlete triad caused a displaced stress fracture of the femoral neck in an adolescent female distance runner.

## Case presentation

**Figure 1:**
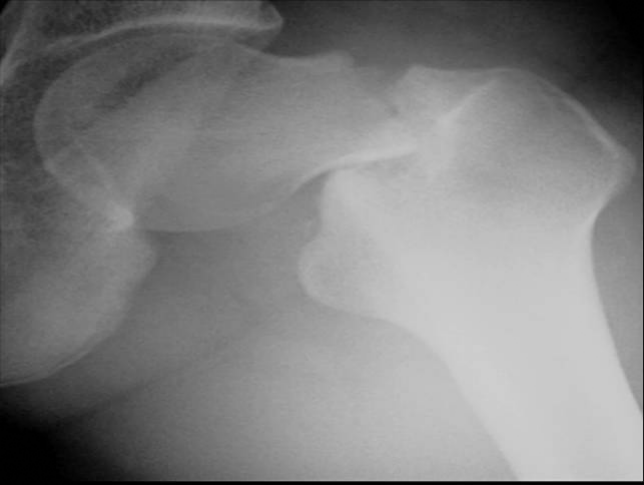
**An anteroposterior X-ray of the left hip showing the femoral neck fracture**. The Devas classification showed that the fracture was a displaced type fracture.

**Figure 2:**
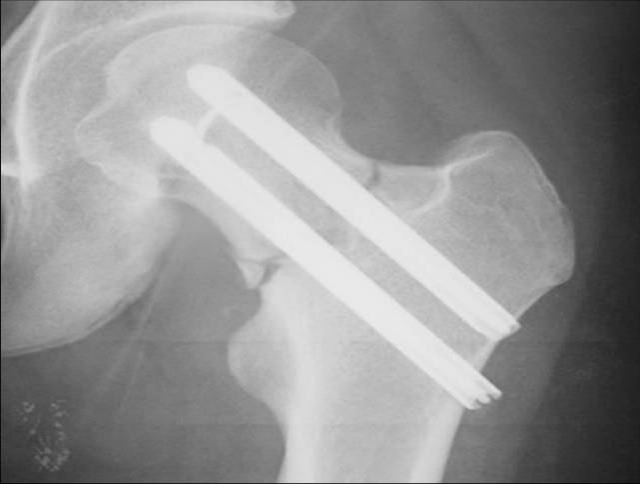
**Postoperative X-ray of the left hip**. The fracture was reduced, and internal fixation was done with the Hansson Pin System^® ^(Stryker).

**Figure 3:**
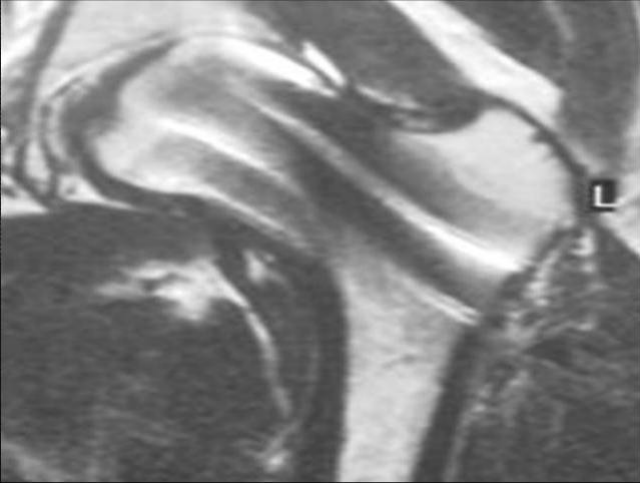
**Follow-up MRI 6 months after surgery**. T1-weighted images demonstrate an area of low signal intensity at the fracture site, but there is no low intensity band in the femoral head.

**Figure 4:**
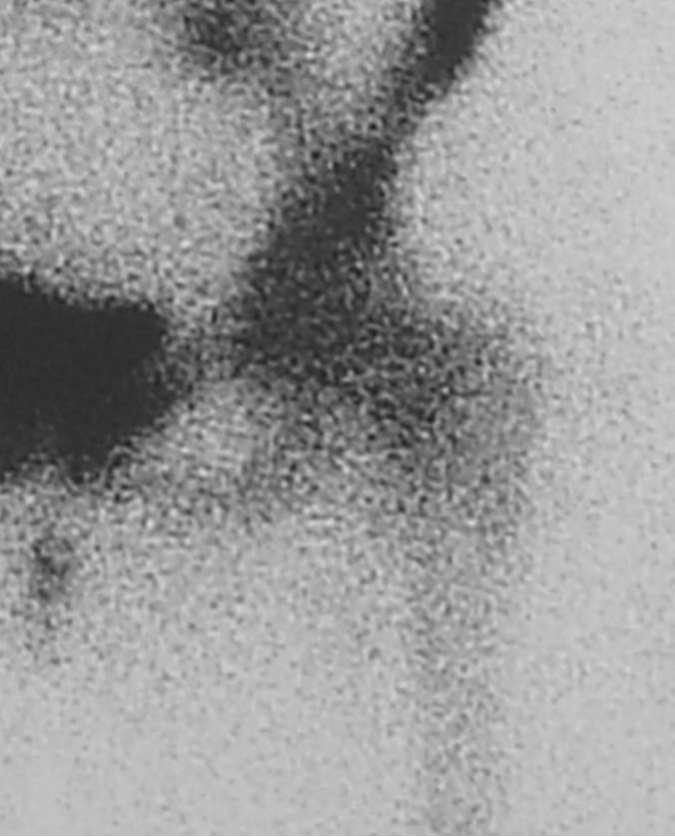
**Follow-up bone scintigraphy 6 months after surgery**. Fracture site and the femoral head are positive, but there is no 'cold-in-hot' appearance in the femoral head.

**Figure 5:**
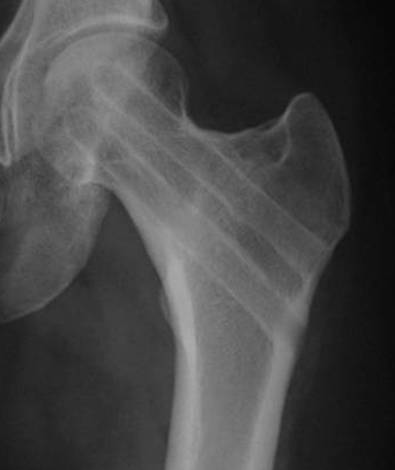
**Anteroposterior X-ray of the left hip 2 years postoperatively**. Bone union is evident.

The patient was a 17-year-old female who had been a long distance runner for five years. Her height was 160 cm, her weight was 47 kg, and her body mass index (BMI) was 18.4. She had run about 210 km a week during her junior high school years and 140 km a week during her high school years on a tartan track. Her running form was the large stride type and her average time was 3 minutes and 40 seconds per kilometer. She had not reached menarche. There are two types of amenorrhea. Primary amenorrhea is the absence of menstruation in a woman by the age of 16 years. Secondary amenorrhea is where an established menstruation has ceased for three months in a woman with a history of regular cyclic bleeding, or nine months in a woman with a history of irregular periods. Her coaches had placed her on a strict diet to keep her weight low since she was 12 years old. In particular, she had never eaten dinner for two weeks before any track meets. She developed pain in the anterior part of the left thigh while training nine days before a prefectural track meet, but she resumed training two days later. Seven days later, she suddenly developed severe pain in the left hip while running, and she was thereafter unable to walk or run. She was taken to the hospital (Department of Orthopaedic Surgery, Kyoto Interdisciplinary Institute Hospital of Community Medicine, Kyoto, Japan) by ambulance. An examination revealed that her left hip was slightly flexed, and she could not move the hip joint actively or passively. Anteroposterior X-rays of the left hip showed a femoral neck fracture (Figure 1). The Devas classification of the fracture was a displaced type [2]. The femoral head was adducted, and the shaft had been displaced upward by 2.5 cm. There was no continuity between the proximal and distal fragments. No obvious signs of sclerosis were seen in the femoral neck. Bone trabeculae were clearly seen in the femoral neck, indicating that there was no bone atrophy. The atrophy of the right femoral neck was classified to be grade 6 according to Singh's index [3], which indicated no bone atrophy. The neck-shaft angle of the right femur was 138° and the alignment of the right lower limb was normal. Her serum biochemistry profile results were all within the normal range: Ca, 9.4 mEq/dl; P 3.7 mEq/dl; and ALP 250 IU/l. Her serum FSH, LH, and estradiol were measured to investigate her primary amenorrhea; the values (FSH, 3.0 mIU/ml; LH, 2.1 mIU/ml; and estradiol, 39.4 pg/ml) all decreased, thus indicating a pituitary dysfunction which thus had caused primary amenorrhea (Table 1). The fracture was easily reduced by skeletal traction. Two days later, the patient had surgery. She was placed in the supine position on a fracture table, and post-reduction alignment was checked using an image intensifier. Internal fixation was performed with the Hansson Pin System^® ^(Stryker) (Figure 2). The patient was started on partial weight-bearing after 1 week; however, weight-bearing was restricted because the varus deformity of the femoral neck shaft angle increased by 3°. The varus deformity was subsequently stabilized. The bone mineral density (BMD) of the left calcaneal bone, which was used to predict of the generalized fracture [4], was evaluated using dual energy X-ray absorptiometry (DEXA) 4 weeks postoperatively. The BMD was 0.870 g/cm$^2$, with a young adult mean of 103%, thus showing no bone loss. Callus formation was identified on X-rays two months postoperatively. The patient was started on full weight-bearing 3 months postoperatively. A plain X-ray of the left hip taken at 6 months confirmed bone union. T1-weighted MRI images obtained at that time showed an area of low signal intensity at the fracture site, but no low intensity band was seen in the femoral head (Figure 3). Bone scintigraphy was positive at the fracture site and the femoral head, but did not show a 'cold-in-hot' appearance in the femoral head (Figure 4). The Hansson Pin System was removed 12 months postoperatively because bone union was obtained and it might have become difficult to remove the implant over time. There was no evidence of necrosis of the femoral head on plain X-ray at 24 months after the injury (Figure 5), and she returned to the same runner level before the injury with no complaints.

**Table 1:** Serum biochemistry profile FSH, LH and estradiol, were all relatively decreased, indicating pituitary dysfunction.

LH	2.1	mIU/ml	(1.8-7.6)
FSH	3	mIU/ml	(5.2-14.4)
Prolactin	11.3	ng/ml	(1.4-14.6)
Estradiol	39.4	pg/ml	(30-400)
Ca	9.4	mEq/dl	(7.8-10.1)
P	3.7	mEq/dl	(3.5-4.5)
ALP	250	IU/l	(122-482)

## Discussion

Stress fractures occur due to repetitive cyclical loading to the same bone site, in contrast to traumatic fractures caused by external force. A stress fracture is the usual injury seen in athletes, particularly in long distance runners. Stress fractures primarily affect the lower extremities [5], with involvement of the tibia in 49%, the tarsals in 25%, and the metatarsals in 8.8% [6]. A femoral neck stress fracture is a rare injury (approximately 1%) [7], and there have been few reports of displaced type femoral neck stress fractures. The primary presenting symptom of a femoral neck stress fracture is usually anterior groin pain. The pain is often exacerbated by training, such as jogging or running, and is relieved by rest. The current case had all of these symptoms.

Exercise-induced amenorrhea was thought to be a normal part of high-intensity training in 1980, and that once stressful training was discontinued, the menstrual cycle would return to normal [8]. However, some female athletes with amenorrhea also have an eating disorder and osteopenia. The American College of Sports Medicine first defined the female athlete triad in 1992, which includes an eating disorder, amenorrhea, and osteoporosis, and stressed that the three components of the triad were associated [1]. One of the mechanisms that is thought to occur in these patients is suppression of the hypothalamic-pituitary-ovarian axis secondary to hypothalamic dysfunction [9,10]. Hypothalamic pulsatile release of gonadotropin releasing hormone (GnRH) decreases both with excessive dieting, which the athlete considers necessary to optimize performance, and the psychological stress caused by external forces, such as coaches, teammates, and parents. Subsequently, the reduced secretion of luteinizing hormone (LH) and follicular stimulating hormone (FSH) from the pituitary gland leads to ovarian suppression, which in turn can lead to hypoestrogenism. The current patient had her caloric intake restricted by her coach since junior high school to optimize her performance and she was under added pressure due to worries about her athletic ranking, her times, and the expectations of others. The mean BMI of an adolescent female athlete has been reported 21.6 or 20.1 [11,12]. Her BMI was relatively low in comparison to these reports. Her FSH was 3.0 mIU/ml, and her LH was 2.1 mIU/ml, which indicated suppression of secretion from the pituitary gland, and her estradiol was 39.4 pg/ml, which indicated ovarian suppression. These results suggest that hypothalamic dysfunction due to physical and psychological factors cause primary amenorrhea.

A long-term hypoestrogenic state due to untreated amenorrhea decreases bone mineral density, which can predispose the patient to stress fractures of the femoral neck or vertebral bodies [13]. The stress fracture in the current patient might have been due to hypoestrogenic state since her the amenorrhea had not been treated even though she had not yet reached menarche at the age of 17 years.

In contrast to the insufficiency fractures seen in postmenopausal women, stress fractures in female athletes frequently occur in the cortical bone of the lower limbs rather than in the cancellous bone [14]. This suggests that osteopenia occurs in the cortical bone in female athletes. The femoral neck stress fracture in this patient may have arisen as the result of a different mechanism than that seen in postmenopausal osteoporosis, since an X-ray of the right femoral neck showed no bone atrophy and the BMD of the cancellous bone was also in the normal range.

Sustained low calorie intake in young athletes undergoing excessive training may pose a risk of increased muscular fatigue, and reduced muscular support of the long bones of the lower extremity [15]. The patient in this report sustained her injury nine days before a prefectural track meet; thus, the muscle fatigue of lower limbs caused by an increase in her training schedule before the meet may have also contributed this fracture.

The fundamental treatment for female athlete triad is weight restoration and a decrease in exercise compatible with energy intake or an associated increase in caloric intake [16]. Hormone replacement therapy (HRT) is often used for the treatment for hypothalamic amenorrhea, however, the effectiveness of HRT on bone mineral density remains controversial [17-19]. HRT in these younger athletes is not routinely recommended. However, a female over the age of 16 with hypothalamic amenorrhea should be given estrogen supplementation [9]. HRT should be administered after bone growth is complete in younger amenorrheic athletes [20]. If a normal menstrual cycle cannot be obtained after an increase in body weight and bone maturation, HRT may thereafter be recommended.

A displaced fracture of the femoral neck leads to a 60% reduction in the patient's activity level in sport and a 30% incidence of avascular necrosis [21]. Magnetic resonance imaging and bone scintigraphy showed no positive signs of femoral head necrosis and bone union was confirmed on plain X-ray in the current patient. Furthermore, the patient returned to her same athletic level prior to injury at 24 months postoperatively, with no clinical symptoms. However, this patient will be needed follow up in the future due to the femoral head necrosis after the femoral neck fracture.

A medical examination for the presence of signs of the female athlete triad by the checking weight, calorie intake and menstrual cycles is crucial to prevent such stress fractures. Athletes as well as their couches or parents need to understand female athlete triad. The fundamental treatment for female athlete triad is to decrease intense exercise and increase daily calorie intake. HRT may be recommended in an athlete in whom the normal menstrual cycle is not restored after an increase in body weight and bone maturation.

## Conclusion

Adolescent female distance runners with amenorrhea or eating disorders are may therefore tend to suffer stress fractures of general bones. In particular, a displaced stress fracture of the femoral neck may cause an irreversible disability, such as femoral head necrosis. A medical examination to check the weight, calorie intake and menstrual cycles is important to prevent such injuries. Athletes as well as their coaches or parents need to understand the female athlete triad.

## Consent

Written informed consent was obtained from the patient for publication of this case report and any accompanying images.

## Competing interests

The authors declare that they have no competing interests.

## Authors' contributions

SO participated in the acquisition of data and drafting the article. YA critically reviewed the drafts for important intellectual content and participated in writing this article. KH, TT and TK have been involved in drafting the manuscript or revising it for important intellectual content. All authors read and approved the final manuscript.
